# Membrane skeleton orchestrates the platelet glycoprotein (GP) Ib‐IX complex clustering and signaling

**DOI:** 10.1002/iub.1559

**Published:** 2016-09-15

**Authors:** Dan Shang, Zuping Zhang, Qian Wang, Yali Ran, Tanner S. Shaw, John N. Van, Yuandong Peng

**Affiliations:** ^1^ Department of Vascular SurgeryUnion Hospital, Tongji Medical College, Huazhong University of Science and TechnologyWuhanHubeiChina; ^2^ Department of Medicine, Cardiovascular Research SectionBaylor College of MedicineHoustonTX; ^3^ Department of Parasitology, School of Basic MedicineCentral South UniversityChangshaChina; ^4^ Department of Medicine, Infectious Disease SectionBaylor College of MedicineHoustonTX; ^5^ Present address: Qian Wang is currently at Department of Dermatology and Venereology of Nan Fang HospitalSouthern Medical UniversityGuangzhouGuangdong Province510515China

**Keywords:** platelet glycoprotein Ib‐IX complex, membrane skeleton, clustering, membrane lipid domain, signaling

## Abstract

Platelet glycoprotein Ib‐IX complex is affixed to the membrane skeleton through interaction with actin binding protein 280 (ABP‐280). We find that removal of the ABP‐280 binding sites in GP Ibα cytoplasmic tail has little impact on the complex clustering induced by antibody crosslinking. However, large truncation of the GP Ibα cytoplasmic tail allows the formation of larger patches of the complex, suggesting that an ABP‐280 independent force may exist. Besides, we observe that the signaling upon GP Ib‐IX clustering is elicited in both membrane lipid domain dependent and independent manner, a choice that relies on how the membrane skeleton interacts with the complex. Our findings suggest a more complex mechanism for how the membrane skeleton regulates the GP Ib‐IX function. © 2016 The Authors IUBMB Life published by Wiley Periodicals, Inc. on behalf of International Union of Biochemistry and Molecular Biology, 68(10):823–829, 2016

## Introduction

The GP Ib‐IX complex is comprised of three type‐I transmembrane polypeptides, GP Ibα, GP Ibβ and GP IX 1. Of which, the extracellular domain of GP Ibα mediates the binding of platelets to subendothelial von Willebrand factor (vWf) and ensures efficient hemostasis 2. Intracelluarly, only GP Ibα can associate with the membrane skeleton of both resting platelets and Chinese Hamster Ovary (CHO) cells through the interaction of its cytoplasmic tail (CT) with the actin binding protein (ABP‐280) 3, 4, 5, 6, 7, 8, 9, 10, 11. A number of investigations have suggested that GP Ibα binding to ABP‐280 facilitates resistance to high shear force upon vWf binding 12, 13, 14 and transmits signals for integrin activation 15, 16. However, several recent investigations have argued against this notion 17, 18. Upon high shear induced vWf binding, platelets can form membrane tethers which originate from the initial discrete adhesion points (DAPs) and later develop multiple secondary DAPs. Abundant amounts of GP Ibα was found on these DAPs 17, 18. It is intriguing, even though membrane tension can eventually be overcome, it does not occur until the hydrodynamic forces reach a certain level (>6,000 s^−1^) 18. Because 1 almost no microfilaments appear in the tethers and DAPs, 2 tethers stretch at a rate faster than the actin can polymerize, and 3 an actin polymerization inhibitor did not prevent the formation of tethers and DAPs, it indicated that the formation of tethers and DAPs do not result from platelet cytoskeletal reorganization, but rather, from the membrane deformation by the pulling force exerted by the clustered GP Ib‐IX/vWf bonds at one single adhesion point 17, 18. Along the same line, the transgenic murine platelets expressing human GP Ibα with the ABP‐280 site removed also formed tethers and membrane debris was deposited on a human vWf‐bound surface at shear rates higher than 5,000 s^−1^ 14. Likewise, in CHO cells expressing the same mutant GP Ibα, not until the shear force reached 40 dyn/cm^2^ or higher could large membrane fragments be pulled off from the cell membrane 11, a level similar to perfusing whole blood at a shear rate greater than 10,000 s^−1^ 19, 20, 21. In comparison, at low shear stresses of 2 to 8 dyn/cm^2^, neutrophils, which are similar in size to CHO cells, can form and break tethers when P‐selectin glycoprotein ligand 1 interacts with immobilized P‐selectin 22, 23, 24. Thus, even though the proposed mechanism of cytoskeletal anchorage through ABP‐280 binding to maintain the GP Ib‐IX‐mediated cell adhesion to immobilized vWf under elevated high shear flow is still valid, it cannot explain why the shear force has to be beyond a certain threshold point in order to overcome the membrane tension when the ABP‐280 binding site is removed or actin polymerization is inhibited. Therefore, it is likely that additional unknown forces exist to hold the GP Ib‐IX complex on cell membranes and to prevent membrane loss at non‐physiological high shear rates below the threshold points (e.g. <5,000 s^−1^).

The specialized glycosphingolipid‐enriched membranes (GEMs) can regulate the GP Ib‐IX function 20, 25, 26, 27, 28, 29, 30. In resting platelets, the GEMs uniformly distributes across the plasma membrane 31. Upon platelet activation by physiological agonists, e.g. immobilized fibrinogen, collagen or thrombin, small GEMs can form large visible aggregates on platelet membranes 26, 32. Even though it remains unclear whether these processes depend on an intact membrane skeleton, it has been reported that these activated platelets lose their discoid shapes, implicating the membrane skeleton may be involved. In the case of the GP Ib‐IX complex, because ABP‐280 can be degraded under high shear flow 33, it is likely that a thus release of the membrane skeletal constraint would facilitate the clustering of the GP Ib‐IX complex and the coalescence of the GEMs upon vWf binding.

In this study, by utilizing K562, a human erythroleukemia cell line, we found that additional forces beyond the ABP‐280‐mediated affixation may exist in regulating the clustering of the GP Ib‐IX complex as well as in the signaling induced in these processes.

## Experimental Procedures

### Antibodies and Chemicals

The following antibodies were used for the immunofluorescent staining of either GP Ibα or the GEMs in K562 cells: mouse monoclonal anti‐human CD42b (clone SZ2) and its FITC conjugated derivatives (Beckman Coulter), Alexa Fluor® 488‐conjugated goat anti‐mouse IgG (H + L) polyclonal secondary antibody (Invitrogen), horseradish peroxidase‐conjugated phospho‐tyrosine mouse monoclonal antibody (P‐Tyr‐100) (Cell Signaling). Alexa Fluor 565‐conjugated recombinant cholera toxin subunit B (CT‐B) lipid raft labeling kit was purchased from Invitrogen. The cholesterol depriving reagent, Methyl‐β‐cyclodextrin (MβCD), was purchased from Sigma.

### Retroviral Constructs and Cell Lines

Retroviral constructs were made by cloning the human cDNAs of GP Ibα, GP Ibβ, and GP IX into: XhoI and EcoRI sites of pMSCV‐puro for GP Ibα, XhoI, and HpaI of pMSCV‐hygromycin for GP Ibβ, and the EcoRI and XhoI sites of pMSCV‐neomycin for GP IX. Site‐directed mutagenesis was performed directly on the GP Ibα construct to either change the amino acids in or truncate the GP Ibα CT. A stable K562 cell line expressing wild type GP Ibβ and GP IX was first generated by hygromycin (400 μg/mL) and neomycin (400 μg/mL) selection and then used as recipient cells for transduction with wild type or mutant GP Ibα viral supernatants.

### Immunofluorescent Staining

GP Ibα‐expressing K562 cells (1 × 10^6^) were washed and resuspended in 20 μL of 0.5% BSA in calcium/magnesium free phosphate‐buffered saline PBS plus 2mM EDTA. The Fc receptor was blocked with 2 μL of FcR blocking reagent (Miltenyi) for 10 min at 4 °C. The SZ2 antibody was directly added to the mixture and diluted with a binding buffer (145 mM NaCl, 5 mM KCl, 4 mM Na_2_HPO_4_, 1 mM MgSO_4_•7H_2_O, 1 mM CaCl_2_, and 10 mM glucose). After incubating for 1 h at room temperature, the cells were washed and then incubated with an Alexa Fluor 488‐labeled goat anti‐mouse secondary antibody to crosslink the GP Ibα antibody. In parallel, the same cells were also stained with a FITC‐conjugated SZ2 antibody. The staining of the GEMs was performed as recommended by the manufacturer where the cells were incubated with Alexa Fluor® 488 conjugated CT‐B for 10 min at 4 °C. Stained cells were fixed in 4% paraformaldehyde prior to fluorescence microscopic analysis.

### Tyrosine Phosphorylation Analysis

Cells were incubated with the SZ2 antibody and then incubated with an unconjugated rabbit anti‐mouse antibody for 60 min at room temperature to crosslink the GP Ibα. Alternatively, cells were treated with 10 mM MβCD at 37 °C for 30 min prior to antibody incubation. Equal numbers of cells were then lysed with reducing laemmli buffer and loaded for SDS‐PAGE gel analysis. The tyrosine phosphorylation was detected by the HRP‐conjugated p‐Tyr‐100 antibody. The loading levels were determined using a β‐tubulin monoclonal antibody (Santa Cruz).

## Results

### Crosslinking of the GP Ibα‐Specific Antibody Clusters GP Ibα

**Figure 1:**
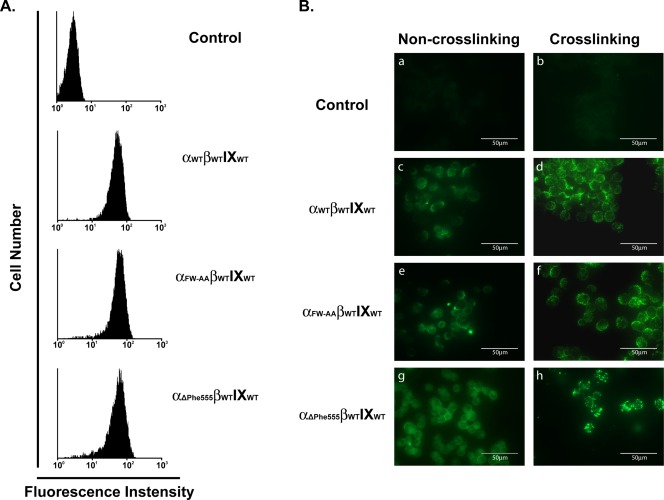
The membrane skeleton restricts the clustering of the GP Ib‐IX complex upon antibody crosslinking. (A) Wild‐type GP Ibα_WT_, ABP‐280‐binding deficient GP Ibα_FW‐AA_ (Phe568Ala and Trp570Ala) and CT‐truncated GP Ibα_ΔPhe555_ were retro‐virally transfected into β_WT_IX_WT_‐expressing K562 cells. Flow cytometry analysis with a phycoerythrin‐labeled anti‐GP Ibα antibody showed that GP Ibα can be efficiently expressed with comparable levels in the βIX‐harboring K562 cells. (B) Cells were first incubated with the mouse anti‐GP Ibα monoclonal antibody (SZ2) or an isotype‐matched mouse antibody, and then left untreated (a, c, e, and g) or treated (b, d, f, and h) by crosslinking with an Alexa Fluor 488–labeled goat anti‐mouse secondary antibody. In the absence of antibody crosslinking, the GP Ibα evenly distributes on all GP Ibα‐expressing cells (c, e, and g) with little sign of GP Ibα clusters in any of these cells, demonstrating that loss of the membrane skeletal constraint does not cause a passive clustering of the surface GP Ibα. In comparison, antibody crosslinking causes the formation of positive GP Ibα‐staining clusters where minor difference in the size or distribution patterns of these clusters were shown between wild‐type GP Ibα_WT_ and ABP‐280‐binding deficient GP Ibα_FW‐AA_ expressing cells (d and f). In sharp contrast, large punctuated GP Ibα‐positive clusters appeared in the CT‐truncated GP Ibα_ΔPhe555_ cells upon antibody crosslinking (h), suggesting that additional force(s) other than the ABP‐280‐mediated membrane skeletal constraint may exist to restrict the movement of the GP Ibα on the cell surface.

It is known that simultaneous mutations of Phe568 and Trp570 to alanines (F_568_A and W_570_A) abolished the binding of human GP Ibα to ABP‐280 14, 34, 35. Here, we made viral constructs and generated the GP Ibβ/GP IX‐harboring K562 cells expressing either wild type GP Ibα_WT_ or the ABP‐280‐binding deficient GP Ibα_FW‐AA_ (Fig. 1A). We first stained the cells with the FITC‐SZ2, and used the IgG isotype matched FITC‐labeled antibody as the control. Consistent with the previous finding that GP Ibα evenly distributes on the platelet surface as detected by gold‐conjugated antibody staining 31, we found that the wild type cells showed uniform distribution of GP Ibα on the cell surface (Fig. 1B, left column, c), whereas the control cells showed little sign of GP Ibα‐positive signals (Fig. 1B, left column, a), indicating that the labeling of GP Ibα on these cells is specific. In contrast, pre‐incubation of these cells with an unconjugated SZ2 antibody followed by a crosslinking of this antibody with an Alexa Fluor 488‐conjugated anti‐mouse antibody produces a distinct pattern where punctuated and clustered GP Ibα‐positive signals are evenly distributed on the cell surfaces (Fig. 1B, right column, d). In comparison, when the mutant cells (GP Ibα_FW‐AA_) are labeled with FITC‐SZ2, we found the distribution pattern was nearly identical between mutant GP Ibα and wild type molecules (Fig. 1B, left column, e), suggesting that a passive coalescence of GP Ibα molecules does not occur when the ABP‐280‐binding mediated membrane skeletal constraint is absent. Nonetheless, to our surprise, we found the distribution pattern was also unaltered in these mutant cells upon antibody crosslinking when compared to the wild type cells (Fig. 1B, right column, f). Because ABP‐280 binding is the only known force capable of restricting the diffusion and clustering of GP Ibα on the cell membrane, our data suggests that 1 clustering of the GP Ib‐IX complex is a ligand‐binding dependent process; and 2 there may be additional force(s) to restrict the clustering in addition to ABP‐280 binding. Furthermore, even though partial deletion of the GP Ibα CT generated inconsistent results regarding the specific sites for ABP‐280 binding 10, 11, 36, larger truncation data has demonstrated that removal of the CT region after residue Ser559 can not only eliminate ABP‐280‐GP Ibα binding but also dissociate the GP Ib‐IX complex from the cell membrane skeleton 7, 10. We therefore made one additional viral construct and established a stable β/IX‐K562 cell lines expressing mutant GP Ibα_ΔPhe555,_ a CT‐truncated GP Ibα with 5 amino acids more that were deleted upstream from the Ser559 residue (Fig. 1A). Upon antibody crosslinking, large patches of GP Ibα‐positive signals, in contrast to that in the GP Ibα_WT_ and GP Ibα_FW‐AA_ cell lines, appeared in the GP Ibα_ΔPhe555_ cells (Fig. 1B, right column, h). In the absence of antibody crosslinking, GP Ibα_ΔPhe555_ evenly distributes on the cell surface with little sign of cluster structures (Fig. 1B, right column, g), a similar pattern that is shown in the wild type and the GP Ibα_FW‐AA_ (Fig. 1B, right column, c and e). Thus, our data suggests that the segment downstream of the Phe555 residue contains structural elements that can restrict GP Ibα movement when the ABP‐280 constraint is absent.

### Clustering of GP Ibα is Accompanied by GEMs Patching

**Figure 2:**
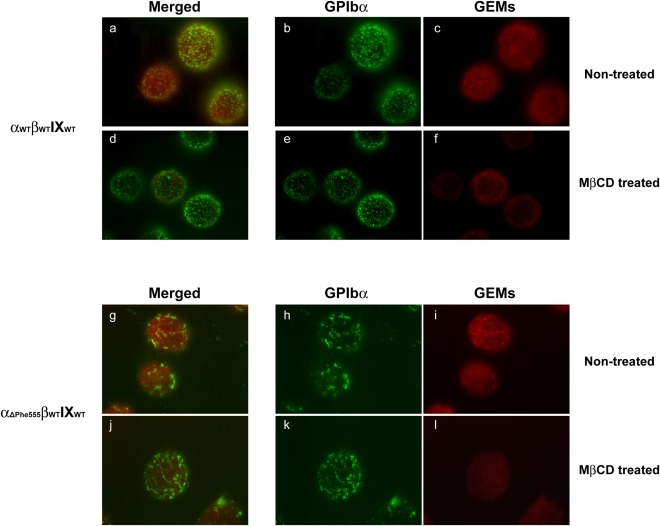
The membrane GEM domain is not essential for antibody‐crosslinking induced GP Ibα clustering. Wild‐type GP Ibα_WT_ and CT‐truncated GP Ibα_ΔPhe555_ were untreated or pretreated with MβCD, a known cholesterol depriving and GEMs disrupting reagent, followed by SZ2 staining and antibody crosslinking, as described in Fig. 1. After fixation, the membrane distribution of the GEM domain was revealed by counterstaining the cells with Alexa Fluor® 488 conjugated CT‐B. In both cells the GP Ibα clusters (green, b, e, h, and k) co‐localized well with the CT‐B patches (red, c, f, i, and l), where all GP Ibα clusters associated with the CT‐B patches (merged, a, d, g, and j). Larger punctuated GEMs patches were formed in the GP Ibα_ΔPhe555_ cells than those in GP Ibα_WT_ cells (c and i), suggesting the GEMs forms patches when GP Ibα clusters. Treatment with MβCD did not affect GP Ibα clustering (e and k) but abolished the GEMs (f and l) and the GP Ibα/GEMs‐colocalizing structures in both cells (merged, d and j), indicating that the clustering of GP Ibα does not depend on the GP Ibα‐associating GEMs, instead, on the presence of the membrane skeletal constraint.

To test if GP Ibα clustering by antibody crosslinking also leads to a simultaneous clustering of GEMs, an important membrane structure for the GP Ib‐IX complex function, we crosslinked GP Ibα on the GP Ibα_WT_ and GP Ibα_ΔPhe555_ cells and then stained the cells with Alexa Fluor 594‐conjugated CT‐B, a GEMs‐specific binding reagent that has high affinity for glycosphingolipid GM1 and lower affinity for other gangliosides 37. We found that in both cell lines the CT‐B patches (Fig. 2, red, c and i) co‐localized well with the GP Ibα clusters (Fig. 2, green, b and h), where all GP Ibα clusters associated with the CT‐B patches and larger punctuated GEMs patches were formed in GP Ibα_ΔPhe555_ cells than those in GP Ibα_WT_ cells (Fig. 2, merged, a and g). Because crosslinking of CT‐B alone in these two cells only showed small GEMs clusters that evenly distribute on the cell membrane (data now shown), our data indicates that the formation of the larger GP Ibα‐cluster/CT‐B‐positive patches in GP Ibα_ΔPhe555_ cells are due to the pre‐clustering of GP Ibα. On the other hand, treatment with MβCD, a known cholesterol depriving and GEMs disrupting reagent, did not affect GP Ibα clustering (Fig. 2, e and k) but abolished the GEMs (Fig. 2, f and l) and the GP Ibα/GEMs‐colocalizing structures in both cells (Fig. 2, d and j), demonstrating that the clustering of GP Ibα does not depend on the GP Ibα‐associating GEMs, instead, on the presence of the membrane skeletal constraint.

### GP Ibα Clustering Induces GEMs‐Dependent and GEMs‐Independent Protein Tyrosine Phosphorylation

**Figure 3:**
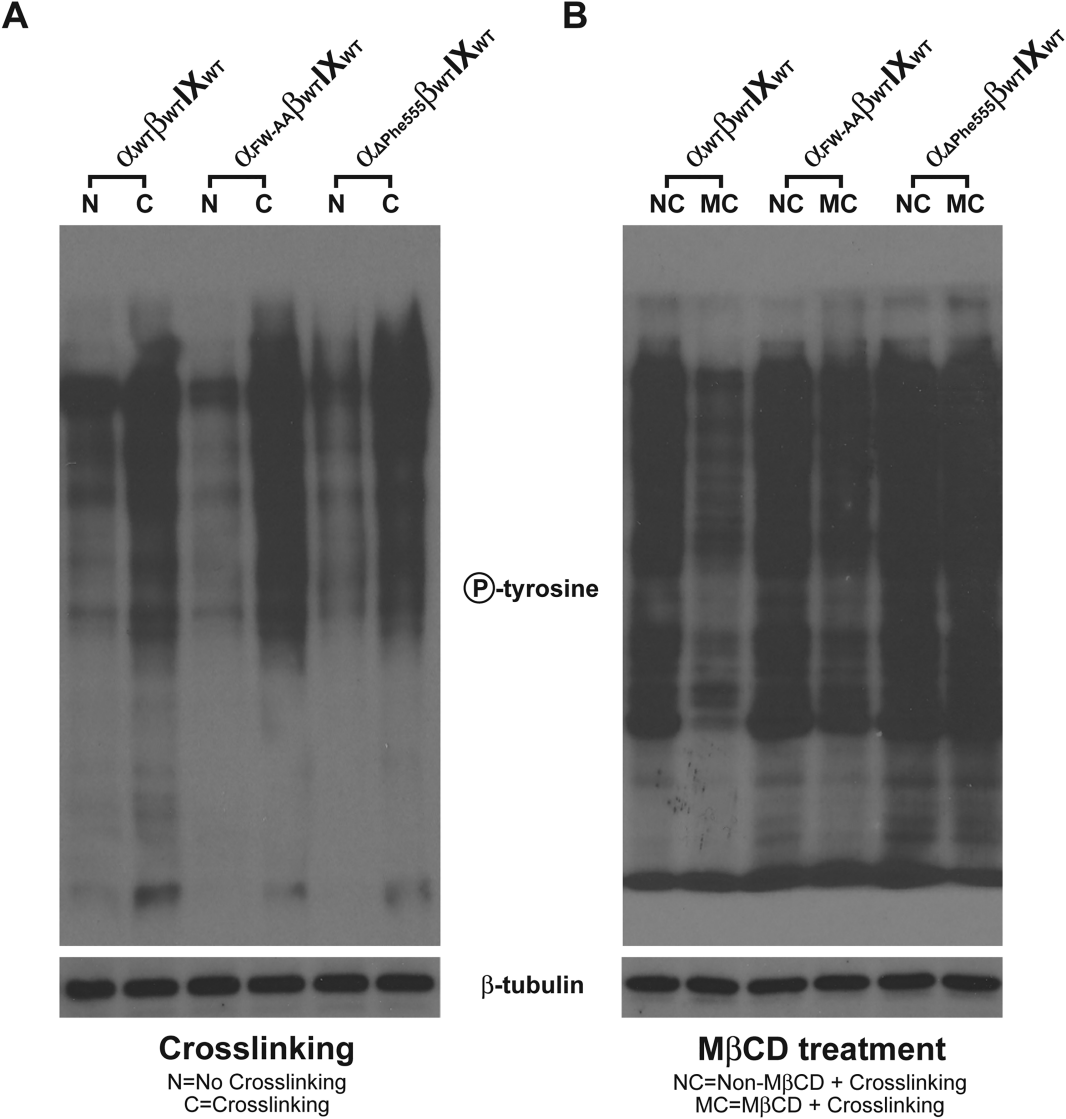
The membrane skeleton and GEM domain regulate the GP Ibα clustering‐induced tyrosine phosphorylation. (A) Wild‐type GP Ibα_WT_, ABP‐280‐binding deficient GP Ibα_FW‐AA_ and CT‐truncated GP Ibα_ΔPhe555_‐expressing K562 cells were first incubated with the SZ2 antibody, and then treated without (N) or with (C) an unconjugated rabbit anti‐mouse antibody. Total protein tyrosine phosphorylation was enhanced upon GP Ibα crosslinking, with the most prominent increase being seen in the lysate of the GP Ibα_ΔPhe555_‐expressing cells. Compared to the wild‐type cells, ABP‐280‐binding deficient GP Ibα_FW‐AA_‐expressing cells do not show a significant increase in the crosslinking‐induced tyrosine phosphorylation. (B) Total protein tyrosine phosphorylation was examined in the cells pretreated without (NC) or with (MC) MβCD after crosslinking of the GP Ibα. In wild‐type and ABP‐280‐deficient cells, MβCD treatment greatly inhibited the crosslinking‐induced tyrosine phosphorylation. In ΔPhe555 cells, however, MβCD treatment had only minor effect, indicating that in these cells the induction of signaling events depends on GP Ibα itself instead of the associating GEMs. These figures are representative of at least three independent experiments.

Upon vWf binding GP Ibα‐expressing platelets or CHO cells showed increased levels of protein tyrosine phosphorylation. To test if GP Ibα clustering due to GP Ibα‐antibody crosslinking causes protein tyrosine phosphorylation, we lysed the GP Ibα‐clustered wild‐type, FW‐AA, and ΔPhe555 cells, and then analyzed for protein tyrosine phosphorylation. As shown in Fig. 3A, clustering of the GP Ib‐IX complex induced protein tyrosine phosphorylation in all three cells. To our surprise, however, disruption of the GEMs by MβCD inhibits the clustering‐induced protein tyrosine phosphorylation only in wild type and GP Ibα_FW‐AA_ (Fig. 3B). In ΔPhe555 cells, MβCD treatment had only minor effect, indicating that in these cells the induced signaling events depends on GP Ibα itself instead of the GP Ibα‐associating GEMs. Thus, our data suggests that GP Ibα clustering can induce tyrosine phosphorylation in a GEMs‐dependent as well as a GEMs‐independent manner, a choice that relies on the presence of ABP‐280‐dependent and independent membrane skeletal constraints.

## Discussion

It has been shown with some molecules, such as TCR, that removal of the membrane skeletal constraint induces a large passive coalescence on the cell membrane. However, this does not occur to the GP Ib‐IX complex in GP Ibα_ΔPhe555_ cells prior to antibody crosslinking. The reasons may lie in several aspects: 1 because the cell membrane skeleton is intact in our cell lines during the staining and treatment, a fence structure underlining the plasma membrane may not be suitable for the movement of large protein complexes (e.g. the GP Ib‐IX complex); 2 associations between the GP Ib‐IX complex with other unknown proteins (some of them may even associate with the membrane skeleton or actin cytoskeleton) may form a larger protein complex to hinder the movement of the GP Ib‐IX complex across the cell membrane. Upon antibody crosslinking, however, the GP Ib‐IX complexes with limited motility can be forced to cluster to different levels (wild‐type, GP Ibα_FW‐AA_, and GP Ibα_ΔPhe555_).

We observed that the well‐characterized ABP‐280‐binding loss‐of‐function GP Ibα_FW‐AA_ can only form small and uniformly distributed clusters upon antibody crosslinking, whereas the mutant GP Ibα with the CT truncated at the Phe555 residue forms larger clusters with nonuniform distribution on the cell surface. This phenotype is quite similar to the formation of large clusters of GP Ib‐IX complexes in the DAPs of platelet tethers, a condition that may represent a complete loss of the membrane skeletal constraint. Thus, our data suggests that the identified CT region after the Phe555 residue may harbor a binding site for some unknown protein(s), in addition to ABP‐280, which can provide additional force(s) to restrict the antibody‐crosslinking induced clustering of the GP Ib‐IX complex. Even though we do not know the exact nature of this protein(s), because high shear stress can break platelet tethers and activate calpain to degrade APB‐280, we speculate that the unidentified protein(s) may be a substrate for calpain as well.

We also observed that the protein tyrosine phosphorylation induced by GP Ibα antibody crosslinking depends on the GEMs only when the membrane skeletal constraint is imposed. In cells where the ABP‐280‐dependent membrane skeletal constraint is eliminated (ΔPhe555), disruption of the GEMs did not alter the clustering‐induced protein tyrosine phosphorylation. Thus, our data suggested that the clustering of the GP Ib‐IX complex can elicit downstream signals in a GEMs dependent and independent manner, which can be regulated by the membrane skeleton. Further investigations will be needed to characterize these two mechanisms, and evaluate the importance in and physiological relevance to the function of the GP Ib‐IX complex.

Taken together, we have demonstrated that the CT of GP Ibα may function in ways that are yet to be defined, 1 associate with unidentified binding partners to strengthen ABP‐280‐mediated membrane skeletal affixation of the GP Ib‐IX complex on the cell membrane, 2 act as an adaptor to recruit signaling molecules through the segment prior to residue Phe555, and 3 the CT portion downstream from residue Phe555 may associate with some unidentified molecules to negatively regulate the signaling that is initiated by the clustering of the CT portion upstream from the Phe555 residue. Future investigations will be needed in these three aspects to unravel these regulatory mechanisms that are potentially important for the function of the GP Ib‐IX complex.
